# Association of Body Weight Variability with Adverse Cardiovascular Outcomes in Patients with Pre-Dialysis Chronic Kidney Disease

**DOI:** 10.3390/nu13103381

**Published:** 2021-09-26

**Authors:** Sang Heon Suh, Tae Ryom Oh, Hong Sang Choi, Chang Seong Kim, Eun Hui Bae, Sue K. Park, Yong-Soo Kim, Yeong Hoon Kim, Kyu Hun Choi, Kook-Hwan Oh, Seong Kwon Ma, Soo Wan Kim, on behalf of the KoreaN Cohort Study for Outcomes in Patients With Chronic Kidney Disease (KNOW-CKD) Investigators

**Affiliations:** 1 Department of Internal Medicine, Chonnam National University Medical School and Chonnam National University Hospital, Gwangju 61469, Korea; medssh1984@gmail.com (S.H.S.); tryeomoh@hanmail.net (T.R.O.); hongsang38@hanmail.net (H.S.C.); laminion@hanmail.net (C.S.K.); baedak76@gmail.com (E.H.B.); 2 Department of Preventive Medicine, Seoul National University College of Medicine, Seoul 03080, Korea; suepark@snu.ac.kr; 3 Cancer Research Institute, Seoul National University, Seoul 03080, Korea; 4 Integrated Major in Innovative Medical Science, Seoul National University College of Medicine, Seoul 03080, Korea; 5 Department of Internal Medicine, College of Medicine, Catholic University of Korea, Seoul 06591, Korea; kimcmc@catholic.ac.kr; 6 Department of Internal Medicine, Busan Paik Hospital, College of Medicine, Inje University, Busan 47392, Korea; yeonghnl@inje.ac.kr; 7 Department of Internal Medicine, College of Medicine, Institute of Kidney Disease Research, Yonsei University, Seoul 03722, Korea; Khchoi6@yuhs.ac; 8 Department of Internal Medicine, Seoul National University Hospital, Seoul 03080, Korea; ohchris@hanmail.net

**Keywords:** all-cause mortality, body weight variability, cardiovascular events, chronic kidney disease

## Abstract

To investigate the association of body weight variability (BWV) with adverse cardiovascular (CV) outcomes in patient with pre-dialysis chronic kidney disease (CKD), a total of 1867 participants with pre-dialysis CKD from Korean Cohort Study for Outcomes in Patients With Chronic Kidney Disease (KNOW-CKD) were analyzed. BWV was defined as the average absolute difference between successive values. The primary outcome was a composite of non-fatal CV events and all-cause mortality. Secondary outcomes were fatal and non-fatal CV events and all-cause mortality. High BWV was associated with increased risk of the composite outcome (adjusted hazard ratio (HR) 1.745, 95% confidence interval (CI) 1.065 to 2.847) as well as fatal and non-fatal CV events (adjusted HR 1.845, 95% CI 1.136 to 2.996) and all-cause mortality (adjusted HR 1.861, 95% CI 1.101 to 3.145). High BWV was associated with increased risk of fatal and non-fatal CV events, even in subjects without significant body weight gain or loss during follow-up periods (adjusted HR 2.755, 95% CI 1.114 to 6.813). In conclusion, high BWV is associated with adverse CV outcomes in patients with pre-dialysis CKD.

## 1. Introduction

Patients with chronic kidney disease (CKD) are likely to have experienced body weight fluctuations. Body weight (BW) loss associated with malnutrition–inflammation is prevalent even before the commencement of renal replacement therapy [1,2] and increases mortality [3]. Several factors are associated with appetite impairment in patients with CKD, which further contributes to protein-energy wasting [4]. Conversely, BW gain associated with excess extracellular fluid is also common in patients with CKD, resulting in accelerated coronary artery calcification [5] and increased all-cause mortality [6]. The prevalent use of diuretics in CKD patients further impose the likelihood of body weight variability (BWV) [7]. As these conditions are not mutually exclusive, it could be assumed that a considerable portion of patients with CKD may experience fluctuations in their BW during the progression of CKD, rather than persistent gain or loss of BW. Nevertheless, the clinical impact of BWV in patients with pre-dialysis CKD has not been established.

BWV is an emerging predictor of adverse cardiovascular (CV) outcomes in various clinical contexts, although its probable association with health outcomes has long been suggested in general population [8]. A prospective cohort study reported that BWV is associated with all-cause mortality, and, in a subgroup with body mass index (BMI) < 25 kg/m^2^ at the baseline, is also associated with increased risks of incident diabetes mellitus (DM) [9,10] and atrial fibrillation [11] in general population. A recent nationwide cohort study reported that BWV is associated with increased risks of myocardial infarction, stroke, and all-cause mortality in patients with type 2 DM [12,13] and in patients with in non-alcoholic fatty liver disease [14]. Fluctuation in BW is associated with a higher rate of cardiovascular (CV) events independent of traditional cardiovascular risk factors in patients with coronary artery disease (CAD) [15,16]. The association between BWV and CV outcomes, however, remains to be elucidated in patients with CKD.

We here investigated the association of BWV with CV outcomes in patients with pre-dialysis CKD. As the patients with CKD are prone to experience BW gain or loss during the course of the disease, we analyzed the association between BWV and longitudinal changes of BW in patients with CKD. We also analyzed the association of BWV with CV outcomes in patients without significant BW gain or loss during follow-up periods.

## 2. Materials and Methods

### 2.1. Study Designs and Data Collection from Participants

**Figure 1:**
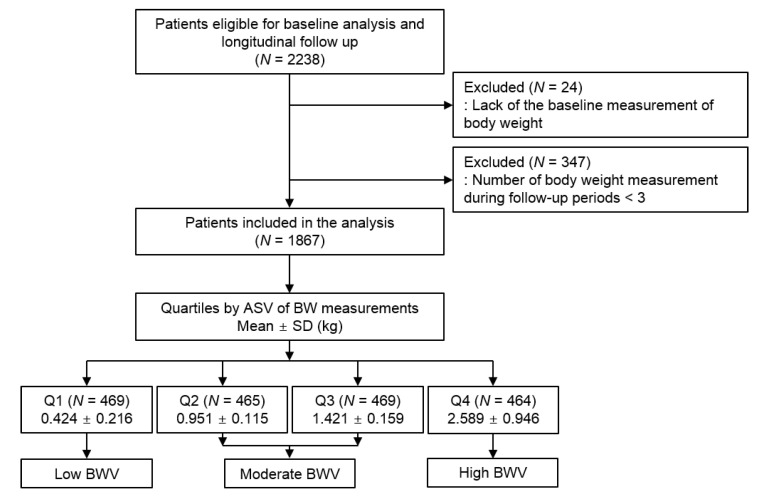
Flow diagram of the study participants. Abbreviations: ASV, average successive variability; BW, body weight; BWV, body weight variability; SD, standard deviation; Q1, 1st quartile; Q2, 2nd quartile; Q3, 3rd quartile; Q4, 4th quartile.

The Korean Cohort Study for Outcomes in Patients With Chronic Kidney Disease (KNOW-CKD) is a nationwide prospective cohort study involving 9 tertiary-care general hospitals in Korea [17]. Korean patients with CKD from stage 1 to pre-dialysis stage 5, who voluntarily provided informed consent were enrolled. The study was conducted in accordance with the principles of the Declaration of Helsinki, and the study protocol was approved by the institutional review boards of participating centers. A total of 2238 subjects were longitudinally followed up. (Figure 1). After excluding those lacking the baseline measurement of BW, and those with the number of body weight measurement during follow-up periods less than three, 1867 subjects were finally included for the analyses. The median follow-up duration was 6.155 years. Demographic information was collected from all eligible participants, including age, gender, comorbid conditions, and medication history (angiotensin-converting enzyme inhibitor/angiotensin II receptor blockers (ACEi/ARBs), diuretics, total number of antihypertensive drugs). Venous samples were collected following overnight fasting, to determine hemoglobin, albumin, total cholesterol, low-density lipoprotein (LDL) cholesterol, high-density lipoprotein (HDL) cholesterol, triglyceride, fasting glucose, high-sensitivity C-reactive protein (hs-CRP), 25(OH) vitamin D and creatinine levels at the baseline. Estimated glomerular filtration rate (eGFR) was calculated by Chronic Kidney Disease Epidemiology Collaboration (CKD-EPI) equation [18]. The urine albumin-to-creatinine ratio (UACR) was measured in random, preferably second-voided, spot urine samples. The 24 h urine protein excretion was also determined.

### 2.2. Determination of BWV

BW was measured at 0, 6, and 12 months and then yearly thereafter up to 8 years. The median number of BW measurement was 6 times. Intra-individual BWV between visits was determined by average successive variability (ASV), defined as the average absolute difference between successive values [9,10,15,16]. The 1st, 2nd and 3rd, and 4th quartiles were defined as low, moderate, and high BWV, respectively (Figure 1).

### 2.3. Estimation of the Rate of Longitudinal BW Change during Follow-Up Periods

The rate of longitudinal BW change for each individual were estimated using a regression model and expressed as the slope (kg/year) [19]. The 1st, 2nd and 3rd, and 4th quartiles were defined as a decrease, maintenance, and increase of BW, respectively (Supplemental Table S1).

### 2.4. Study Outcomes

The primary outcome was a composite of non-fatal CV events and all-cause mortality. Raw numbers for the composite outcome by enrollment sites subgroups are summarized in Supplemental Tables S2 and S3, respectively. Secondary outcomes were fatal and non-fatal CV events and all-cause mortality. CV events, either fatal or non-fatal, included any coronary artery event (unstable angina, myocardial infarction, or coronary intervention/surgery), hospitalization for heart failure, ischemic or hemorrhagic stroke, incident peripheral arterial disease, and symptomatic arrhythmia.

### 2.5. Statistical Analysis

Continuous variables were expressed as mean ± standard deviation or median [interquartile range]. Categorical variables (e.g., current smoking status) were expressed as number of participants and percentage. For descriptive analyses, Student’s T-test or one-way analysis of variance and χ^2^ test were used for continuous and categorical variables, respectively. The correlation between the BWV and BW slope was established by curve estimation regression analysis. Multinomial logistic regression models were analyzed to address the association between BWV and longitudinal BW change, where the models were adjusted for age, gender, Charlson comorbidity index, history of DM, smoking history, BMI, systolic blood pressure (SBP), diastolic blood pressure (DBP), medications (ACEi/ARBs, diuretics, total number of antihypertensive drugs), hemoglobin, albumin, HDL-cholesterol, triglycerides, fasting serum glucose, hs-CRP, 25(OH) vitamin D levels, eGFR, and 24 h urine protein. The results of multinomial logistic regression models were presented as odd ratios (ORs) and 95% confidence intervals (CIs). To assess the association between BWV and the outcomes, Cox proportional hazard regression models were analyzed. Patients lost to follow-up were censored at the date of the last visit. We adjusted age, gender, Charlson comorbidity index, history of DM, smoking history, BMI, SBP, DBP, medications (ACEi/ARBs, diuretics, total number of antihypertensive drugs), hemoglobin, albumin, HDL-cholesterol, triglycerides, fasting serum glucose, hs-CRP, 25(OH) vitamin D levels, eGFR, and 24 h urine protein. The results of Cox proportional hazard models were presented as hazard ratios (HRs) and 95% CIs. Statistical significance was defined as *p* < 0.05. Data were analyzed using IBM SPSS statistical analysis software for Windows, version 22.0 (IBM Corp).

## 3. Results

### 3.1. Baseline Characteristics

The baseline characteristics of study participants were described by BWV (Table 1). The mean age was highest in subjects with low BWV, and lowest in subjects with high BWV. The gender distribution was not significantly different among the groups. Charlson comorbidity index was higher in subjects with high BWV. Detailed presentation of Charlson Comorbidity Index components by BWV is summarized in Supplement Table S4, which revealed that the frequency of cerebrovascular disease and diabetes with organ damage was significantly higher in the subjects with high BWV. The frequency of the subjects with previous medical history of DM was highest in the subjects with high BWV, and lowest in the subjects with low BWV. The frequencies of other medical history, such as CAD and arrhythmia were not significantly different among the groups. Smoking status did not differ across the groups either. BMI, waist circumference, and SBP were highest in the subjects with high BWV, and lowest in the subjects with low BWV. The subjects with high BWV were significantly likely to take no less than 3 antihypertensive drugs at the baseline. Serum albumin level was highest in the subjects with moderate BWV. Serum triglyceride and fasting glucose levels were highest in the subjects with high BWV, and lowest in the subjects with low BWV. Conversely, 25(OH) vitamin D level was highest in subjects with low BWV, and lowest in subjects with high BWV. The 24 h urine protein and UACR in random urine were lowest in in the subjects with moderate BWV. Hemoglobin, total cholesterol, LDL-cholesterol, HDL-cholesterol, hs-CRP levels were not significantly different among the groups. In contrast, eGFR was highest in the subjects with high BWV, and lowest in the subjects with low BWV. Accordingly, the distribution of CKD stages across the groups was significantly different. Collectively, high BWV was in large associated with unfavorable clinical features, with an exception of eGFR, which could be attributed to significantly higher BMI in subjects with high BWV and a relevant limitation of creatinine-based estimation of glomerular filtration rate.

### 3.2. High BWV Is Associated with Adverse CV Outcomes in Patients with Pre-Dialysis CKD

**Figure 2:**
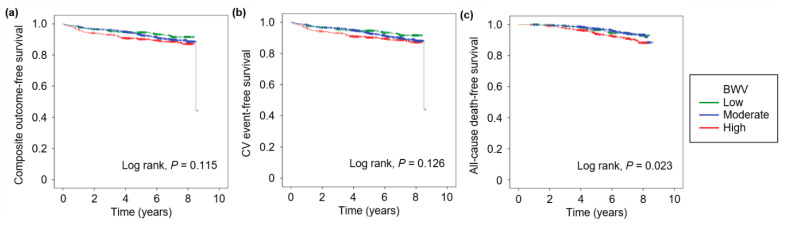
Kaplan–Meier survival curve for the outcomes by BWV. The probability of composite outcome- (**a**), fatal and non-fatal CV event- (**b**), and all-cause death- (**c**) free survivals by BW variability. *p* values by Log rank test. Abbreviations: BWV, body weight variability.

To address the association of BWV and the study outcomes, Kaplan–Meier survival was analyzed by BWV (Figure 2). The survival curves for the composite outcome (Log rank, *p* = 0.115) and fatal and non-fatal CV events (Log rank, *p* = 0.126) revealed no significant differences among the groups by BWV, whereas the all-cause death-free survival significantly differs among the groups (Log rank, *p* = 0.023). To determine whether BWV is independently associated with CV outcomes in patients with pre-dialysis CKD, Cox proportional hazard regression models were analyzed. (Table 2). Compared to the subjects with moderated BWV, those with high BWV were associated with increased risks of the composite outcome (adjusted HR 1.738, 95% CI 1.065 to 2.847, *p* = 0.027), fatal and non-fatal CV events (adjusted HR 1.845, 95% CI 1.136 to 2.996, *p* = 0.013), and all-cause mortality (adjusted HR 1.861, 95% CI 1.101 to 3.145, *p* = 0.020). In the subgroup analyses (Table 3), high BWV was associated with the composite outcome in the subjects with age ≥ 60 years (adjusted HR 2.361, 95% CI 1.139 to 4.897, *p* = 0.021), medical history of DM (adjusted HR 2.199, 95% CI 1.091 to 4.431, *p* = 0.028), and total number of antihypertensive drugs ≥ 3 (adjusted HR 6.172, 95% CI 1.394 to 27.333, *p* = 0.017). Interestingly, both low (adjusted HR 2.542, 95% CI 1.072 to 6.024, *p* = 0.034) and high (adjusted HR 2.545, 95% CI 1.181 to 5.486, *p* = 0.017) BWV were associated with the composite outcomes in those without use of diuretics. Low BWV was also associated with the composite outcomes in those with UACR < 300 mg/g (adjusted HR 3.509, 95% CI 1.028 to 9.105, *p* = 0.045). The association of high BWV with fatal and non-fatal CV events (Supplemental Table S5) were also significant in the subjects with age ≥ 60 years (adjusted HR 2.191, 95% CI 1.054 to 4.557, *p* = 0.036), male gender (adjusted HR 1.865, 95% CI 1.020 to 3.412, *p* = 0.043), medical history of DM (adjusted HR 2.300, 95% CI 1.147 to 4.612, *p* = 0.019), BMI ≥ 25 kg/m^2^ (adjusted HR 3.048, 95% CI 1.181 to 7.869, *p* = 0.021), total number of antihypertensive drugs ≥ 3 (adjusted HR 7.993, 95% CI 2.145 to 29.779, *p* = 0.002), and eGFR < 45 mL/min./1.73 m^2^ (adjusted HR 2.195, 95% CI 1.051 to 4.582, *p* = 0.036). Only high BWV were associated with the fatal and non-fatal CV events in those without use of diuretics (adjusted HR 2.515, 95% CI 1.165 to 5.428, *p* = 0.019). In the subgroup analyses for all-cause mortality (Supplemental Table S6), high BWV was associated with increased risk of all-cause mortality only in the subjects with age ≥ 60 years (adjusted HR 2.438, 95% CI 1.324 to 4.499, *p* = 0.004). Taken together, high BWV is significantly associated with adverse CV outcomes in patients with pre-dialysis CKD.

### 3.3. High BWV Is Associated with Adverse CV Outcomes Even in Patients without BW Gain or Loss during Follow-up Periods

To determine the association between BWV and the rate of longitudinal BW change, multinomial logistic regression models were analyzed (Supplemental Table S7), which demonstrated a robust correlation of high BWV with both BW decrease (adjusted OR 2.310, 95% CI 1.661 to 3.213, *p* < 0.001) and BW increase (adjusted OR 2.642, 95% CI 1.912 to 3.652, *p* < 0.001). Inversely, low BWV was associated with reduced risks of either BW decrease (adjusted OR 0.429, 95% CI 0.300 to 0.614, *p* < 0.001) or BW increase (adjusted OR 0.541, 95% CI 0.384 to 0.763, *p* < 0.001). However, the scatter plot for the correlation of the rate of longitudinal BW change and BWV (Supplemental Figure S1) visualized that a substantial portion of the subjects experience only a limited range of longitudinal BW change, but concurrently show visit-to-visit BWV (i.e., scatter plots condensed in the midline with a distribution along the Y-axis direction). Therefore, we decided to unveil the association of high BWV with adverse CV outcomes in the subjects without BW gain or loss (i.e., the subjects with BW maintenance) during follow-up periods (*n* = 930). The analyses of Cox proportional hazard regression models (Table 4) demonstrated that high BWV is associated with increased risk of fatal and non-fatal CV events in patients with BW maintenance during follow-up periods (adjusted HR 2.755, 95% CI 1.114 to 6.813, *p* = 0.028), suggesting that fluctuations in BW impose an independent risk of CV events even in the absence of longitudinal BW change.

To figure out the impact of the baseline BMI in the subjects with high BWV, the subjects with high BWV were further divided into those with BMI 20 to 24.9 kg/m^2^ (i.e., near normal BMI) vs. those with BMI < 20 kg/m^2^ or ≥r/mesds ^2^ (i.e., relatively underweight or obese) (Supplemental Table S8). Intriguingly, only the subjects with high BWV and BMI < 20 kg/m^2^ or ≥r/mectsn^2^ were associated with increased risk of the composite outcome (adjusted HR 2.149, 95% CI 1.107 to 4.173, *p* = 0.024), fatal and non-fatal CV events (adjusted HR 2.286, 95% CI 1.180 to 4.426, *p* = 0.014), and all-cause mortality (adjusted HR 2.124, 95% CI 1.101 to 4.095, *p* = 0.025), indicating a significant impact of the baseline BMI in addition to BWV on the prognosis of the patients with pre-dialysis CKD.

## 4. Discussion

In the present study, we discovered that high BWV is significantly associated with adverse CV outcomes in patients with pre-dialysis CKD. We also demonstrated that BWV is associated with longitudinal of BW gain or loss in patients with CKD. Importantly, we proved that high BWV is associated with adverse CV outcomes, even in patients without significant BW gain or loss during follow-up periods.

In the current study, we found that BWV is associated with both longitudinal BW gain and loss in patients with CKD, rather than a unidirectional association toward BW gain or BW loss. This suggests the multifaceted nature of the progression in the body composition during the course of CKD. Indeed, a recent cohort study reported that both BW gain and loss are associated with adverse outcomes in patients with pre-dialysis CKD [19]. Meanwhile, in the present study, we primarily highlighted on the visit-to-visit fluctuation in BW, rather than longitudinal trends in BW, provided that several events that promote BW gain or loss may differ among each follow-up visit, in which case the BWV should be high, even though the BW slope might be blunted. Therefore, a potential strength of BWV over longitudinal BW change may be a sensitive detection of vulnerable subjects who are at high risk of CV events. In this context, it is of interest to note that high BWV is associated with adverse CV outcomes, even in patients with BW maintenance during follow-up periods, as this suggests a prognostic impact of BWV independent of longitudinal BW change.

It is intriguing that, although high BWV was robustly associated adverse outcomes, low BWV was also associated with high risk of the composite outcome in certain clinical contexts (e.g., urine ACR < 300 mg/g). We speculate that this results from the complex nature of homeostasis in body composition during the course of CKD, which may justify BWV to some degree. Indeed, in the present study, the risks of adverse outcomes were almost consistently lowest in subjects with ‘moderate’, but not ‘low’, BWV. The precise mechanism of how a moderate, not low, degree of BWV predicts the best outcomes in patients with pre-dialysis CKD should be further elucidated.

The mechanism of how BWV is associated with adverse CV outcomes is another remaining question. One possible explanation is the association of BWV and coronary artery calcification (CAC), although the association has not been validated yet. Previous studies reported that central adiposity is strongly associated with CAC in general population [20] and in CKD patients [21,22]. Inversely, malnutrition–inflammation is associated with a higher CAC score in diabetic CKD patients [23], as well as in patients with chronic dialysis [24,25]. As BWV is a sensitive read-out of conditions associated with BW gain or loss in CKD population, we speculate that BWV may predict the risk of CAC, delineating its association with adverse CV outcomes. Another explanation is the association of BWV and heart failure (HF). It has been reported that the fluctuations in BW [26] or anthropometric indices [27], such as BMI and the waist-to-hip ratio, and BW and is associated with increased mortality in patients with HF. As the renal function of the subjects in those studies is relatively reserved, the association of BWV and HF should be further evaluated.

There are a number of limitations in this study. First, we are not able to clarify the causal relationship between high BWV and adverse CV outcomes, because of the observational nature of the current study. Second, despite the clear impact of high BWV on the adverse CV outcomes, the precise mechanism should be further addressed. Third, as this cohort study enrolled only ethnic Koreans, a precaution is required to extrapolate the data in the present study to other populations. Fourth, there is a potential risk of multiple testing burden in the current study, as the multiple outcomes have been analyzed.

In conclusion, we report that high BWV is significantly associated with adverse CV outcomes in patients with pre-dialysis CKD. Our results suggest that BWV is associated with longitudinal of BW gain or loss in patients with CKD, while high BWV is associated with adverse CV outcomes, even in patients without BW gain or loss during follow-up periods.

## Figures and Tables

**Table 1:** Baseline characteristics of study participants by BWV.

		BWV		
	Low	Moderate	High	*p* Value
Follow-up duration (year)	5.510 ± 2.025	6.024 ± 1.721	6.024 ± 1.785	<0.001
Age (year)	54.889 ± 10.969	54.035 ± 11.603	51.442 ± 13.726	<0.001
Male	278 (59.3)	556 (59.5)	299 (64.4)	0.161
Charlson comorbidity index				0.001
0–3	360 (76.8)	710 (76.0)	313 (67.5)	
≥4	109 (23.2)	224 (24.0)	151 (32.5)	
DM	125 (26.7)	280 (30.0)	187 (40.3)	<0.001
CAD	21 (4.5)	51 (5.5)	35 (7.5)	0.365
Arrhythmia	13 (2.8)	17 (1.8)	14 (3.0)	0.558
Current smoking	68 (14.5)	143 (15.3)	78 (16.8)	0.609
BMI (kg/m^2^)	24.031 ± 3.029	24.266 ± 3.157	25.733 ± 3.787	<0.001
WC (cm)	77.283 ± 26.828	83.284 ± 18.933	85.848 ± 22.309	<0.001
SBP (mmHg)	126.486 ± 14.885	125.775 ± 15.619	128.358 ± 17.207	0.016
DBP (mmHg)	77.348 ± 10.213	76.085 ± 11.071	76.862 ± 12.002	0.111
Medications				
ACEi/ARBs	408 (87.0)	809 (86.6)	401 (86.4)	0.966
Diuretics	320 (68.2)	266 (28.5)	305 (65.7)	0.075
Number of anti-HTN drugs ≥ 3	115 (24.5)	690 (26.1)	161 (34.7)	0.001
Laboratory findings				
Hemoglobin (g/dL)	12.862 ± 2.044	13.098 ± 1.916	12.926 ± 1.983	0.074
Albumin (g/dL)	4.158 ± 0.361	4.240 ± 0.380	4.219 ± 0.404	0.001
Total cholesterol (mg/dL)	173.271 ± 39.409	173.460 ± 34.988	175.320 ± 38.791	0.582
LDL cholesterol (mg/dL)	95.763 ± 31.183	69.189 ± 29.968	96.839 ± 31.456	0.866
HDL cholesterol (mg/dL)	49.246 ± 15.650	50.220 ± 15.404	48.568 ± 14.762	0.148
Triglyceride (mg/dL)	153.421 ± 95.966	152.346 ± 96.268	169.508 ± 105.656	0.007
Fasting glucose (mg/dL)	107.190 ± 38.519	108.214 ± 32.835	116.061 ± 47.564	<0.001
hsCRP (mg/dL)	0.500 [0.300, 1.400]	0.600 [0.200, 1.593]	0.700 [0.300, 2.000]	0.220
25(OH) vitamin D (ng/dL)	18.953 ± 9.220	18.353 ± 7.542	16.456 ± 6.427	<0.001
24 h urine protein (mg/dL)	557.500 [182.300, 1517.000]	454.800 [147.250, 1086.750]	562.800 [179.625, 1568.700]	0.003
Urine ACR (mg/g Cr)	348.798 [77.354, 1023.856]	299.154 [56.893, 772.399]	326.071 [92.807, 1143.938]	<0.001
eGFR (mL/min/1.73 m^2^)	48.549 ± 28.254	52.541 ± 29.456	53.178 ± 31.090	0.003
CKD stages				0.036
Stage 1	59 (12.6)	168 (18.0)	89 (19.2)	
Stage 2	90 (19.2)	202 (21.6)	88 (19.0)	
Stage 3a	88 (18.8)	167 (17.9)	76 (16.4)	
Stage 3b	104 (22.2)	212 (22.7)	97 (20.9)	
Stage 4	107 (22.8)	158 (16.9)	100 (21.6)	
Stage 5	21 (4.5)	27 (2.9)	14 (3.0)	

Values for categorical variables are given as number (percentage); values for continuous variables, as mean ± standard deviation or median (interquartile range). Abbreviations: ACEi, angiotensin-converting enzyme inhibitor; ACR, albumin-to-creatinine ratio; ARB, angiotensin receptor blocker; BMI, body mass index; CAD, coronary artery disease; CCI, Charlson comorbidity index; CKD, chronic kidney disease; DBP, diastolic blood pressure; DM, diabetes mellitus; eGFR, estimated glomerular filtration rate; hsCRP, high-sensitivity C-reactive protein; SBP, systolic blood pressure; WC, waist circumference.

**Table 2:** Cox proportional hazards regression of BWV for the outcomes.

		Unadjusted		Adjusted	
		HR (95% CIs)	*p* Value	HR (95% CIs)	*p* Value
Composite outcome	Low BWV	1.236 (0.743, 2.057)	0.414	1.444 (0.790, 2.642)	0.233
	Moderate BWV	Reference		Reference	
	High BWV	1.789 (1.195, 2.679)	0.005	1.738 (1.065, 2.847)	0.027
Fatal and non-fatal CV events	Low BWV	1.283 (0.771, 2.133)	0.337	1.593 (0.882, 2.884)	0.123
	Moderate BWV	Reference		Reference	
	High BWV	1.827 (1.221, 2.735)	0.003	1.845 (1.136, 2.996)	0.013
All-cause mortality	Low BWV	1.008 (0.543, 1.870)	0.980	0.930 (0.493, 1.753)	0.822
	Moderate BWV	Reference		Reference	
	High BWV	1.702 (1.043, 2.776)	0.033	1.861 (1.101, 3.145)	0.020

Models were adjusted for age, gender, Charlson comorbidity index, history of DM, smoking history, BMI, SBP, DBP, Medications (ACEi/ARBs, diuretics, number of antihypertensive drugs), hemoglobin, albumin, HDL-cholesterol, triglycerides, fasting serum glucose, hs-CRP, 25(OH) vitamin D levels, eGFR, and 24 h urine protein. Abbreviations: BWV, body weight variability; CV, cardiovascular; CI, confidence interval.

**Table 3:** Cox proportional hazards regression of BWV for the composite outcome in various subgroups.

		Unadjusted		Adjusted	
		HR (95% CIs)	*p* Value	HR (95% CIs)	*p* Value
Age < 60 years	Low BWV	1.811 (0.814, 4.030)	0.146	0.595 (0.183, 1.938)	0.204
	Moderate BWV	Reference		Reference	
	High BWV	1.675 (0.879, 3.195)	0.117	0.782(0.265, 2.302)	0.782
Age ≥ 60 years	Low BWV	1.057 (0.541, 2.065)	0.870	1.048 (0.429, 2.559)	0.919
	Moderate BWV	Reference		Reference	
	High BWV	2.089 (1.234, 3.539)	0.006	2.361 (1.139, 4.897)	0.021
Male	Low BWV	1.853 (0.966, 3.554)	0.064	1.512 (0.6323, 3.614)	0.353
	Moderate BWV	Reference		Reference	
	High BWV	1.508 (0.931, 2.440)	0.095	1.723 (0.926, 3.204)	0.086
Female	Low BWV	0.720 (0.302, 1.719)	0.460	0.275 (0.052, 1.438)	0.126
	Moderate BWV	Reference		Reference	
	High BWV	2.606 (1.187, 5.720)	0.017	0.271 (0.049, 1.490)	0.133
CCI ≤ 3	Low BWV	0.883 (0.395, 1.978)	0.763	0.879 (0.287, 2.695)	0.821
	Moderate BWV	Reference		Reference	
	High BWV	2.242 (1.231, 4.083)	0.008	1.910 (0.812, 4.496)	0.138
CCI ≥ 4	Low BWV	1.781 (0.896, 3.542)	0.100	3.855 (1.373, 10.823)	0.010
	Moderate BWV	Reference		Reference	
	High BWV	1.499 (0.841, 2.670)	0.169	1.364 (0.589, 3.157)	0.469
DM (−)	Low BWV	1.164 (0.539, 2.513)	0.700	2.012 (0.659, 6.143)	0.220
	Moderate BWV	Reference		Reference	
	High BWV	1.952 (1.002, 3.800)	0.049	1.429 (0.531, 3.842)	0.480
DM (+)	Low BWV	1.304 (0.654, 2.600)	0.451	2.169 (0.762, 6.174)	0.147
	Moderate BWV	Reference		Reference	
	High BWV	1.620 (0.947, 2.769)	0.078	2.199 (1.091, 4.431)	0.028
BMI < 25 (kg/m^2^)	Low BWV	1.452 (0.785, 2.687)	0.235	2.175 (0.953, 4.965)	0.065
	Moderate BWV	Reference		Reference	
	High BWV	1.891 (1.072, 3.335)	0.028	1.743 (0.813, 3.739)	0.153
BMI ≥ 25 (kg/m^2^)	Low BWV	0.898 (0.341, 2.367)	0.829	1.203 (0.283, 5.125)	0.802
	Moderate BWV	Reference		Reference	
	High BWV	1.578 (0.863, 2.884)	0.138	2.666 (0.938, 7.579)	0.066
Diuretics (−)	Low BWV	1.789 (0.928, 3.447)	0.082	2.542 (1.072, 6.024)	0.034
	Moderate BWV	Reference		Reference	
	High BWV	3.037 (1.659, 5.558)	<0.001	2.545 (1.181, 5.486)	0.017
Diuretics (+)	Low BWV	0.824 (0.353, 1.921)	0.653	0.531 (0.148, 1.914)	0.334
	Moderate BWV	Reference		Reference	
	High BWV	1.283 (0.699, 2.355)	0.422	0.956 (0.398, 2.296)	0.919
Number of anti-HTN drugs ≤ 2	Low BWV	1.374 (0.771, 2.450)	0.281	1.822 (0.869, 3.821)	0.112
	Moderate BWV	Reference		Reference	
	High BWV	1.641 (0.983, 2.738)	0.058	1.670 (0.838, 3.328)	0.145
Number of anti-HTN drugs ≥ 3	Low BWV	0.640 (0.187, 2.187)	0.477	1.451 (0.159, 13.227)	0.741
	Moderate BWV	Reference		Reference	
	High BWV	2.374 (1.199, 4.704)	0.013	6.172 (1.394, 27.333)	0.017
eGFR ≥ 45 mL/min/1.73 m^2^	Low BWV	1.641 (0.624, 4.314)	0.315	0.282 (0.060, 1.326)	0.109
	Moderate BWV	Reference		Reference	
	High BWV	1.956 (1.049, 3.649)	0.035	2.066 (0.855, 4.991)	0.107
eGFR < 45 mL/min/1.73 m^2^	Low BWV	1.130 (0.609, 2.097)	0.698	1.679 (0.754, 3.741)	0.205
	Moderate BWV	Reference		Reference	
	High BWV	1.822 (1.058, 3.318)	0.031	1.626 (0.775, 3.413)	0.199
Random urine ACR < 300 mg/g	Low BWV	1.603 (0.734, 3.501)	0.237	3.059 (1.028, 9.105)	0.045
	Moderate BWV	Reference		Reference	
	High BWV	2.150 (1.154, 4.006)	0.016	2.308 (0.977, 5.454)	0.057
Random urine ACR ≥ 300 mg/g	Low BWV	0.967 (0.485, 1.926)	0.924	0.964 (0.380, 2.447)	0.939
	Moderate BWV	Reference		Reference	
	High BWV	1.464 (0.833, 2.570)	0.185	1.554 (0.690, 3.500)	0.287

Models were adjusted for age, gender, Charlson comorbidity index, history of DM, smoking history, BMI, SBP, DBP, Medications (ACEi/ARBs, diuretics, number of antihypertensive drugs), hemoglobin, albumin, HDL-cholesterol, triglycerides, fasting serum glucose, hs-CRP, 25(OH) vitamin D levels, eGFR, and 24 h urine protein. Abbreviations: ACR, Albumin-to-creatinine ratio; BMI, body mass index; CCI, Charlson comorbidity index; CI, confidence interval; DM, diabetes mellitus; HTN, hypertension; eGFR, estimated glomerular filtration rate.

**Table 4:** Cox proportional hazards regression of BWV for the outcomes in subjects with BW maintenance during follow-up periods.

		Unadjusted		Adjusted	
		HR (95% CIs)	*p* Value	HR (95% CIs)	*p* Value
Composite outcome	Low BWV	1.580 (0.733, 3.406)	0.243	1.878 (0.701, 5.029)	0.210
	Moderate BWV	Reference		Reference	
	High BWV	1.754 (0.872, 3.528)	0.115	2.239 (0.816, 6.148)	0.118
Fatal and non-fatal CV events	Low BWV	1.762 (0.846, 3.669)	0.130	2.430 (0.958, 6.166)	0.062
	Moderate BWV	Reference		Reference	
	High BWV	1.838 (0.913, 3.698)	0.088	2.755 (1.114, 6.813)	0.028
All-cause mortality	Low BWV	1.729 (0.716, 4.179)	0.224	1.530 (0.580, 4.037)	0.390
	Moderate BWV	Reference		Reference	
	High BWV	1.377 (0.438, 4.324)	0.584	1.111 (0.309, 3.999)	0.871

Models were adjusted for age, gender, Charlson comorbidity index, history of DM, smoking history, BMI, SBP, DBP, Medications (ACEi/ARBs, diuretics, number of antihypertensive drugs), hemoglobin, albumin, HDL-cholesterol, triglycerides, fasting serum glucose, hs-CRP, 25(OH) vitamin D levels, eGFR, and 24 h urine protein. Abbreviations: CI, confidence interval.

## Data Availability

Not applicable.

## Supplementary Materials

The following are available online at https://www.mdpi.com/article/10.3390/nu13103381/s1, Table S1: Baseline characteristics of study participants by BW slope. Table S2: Raw numbers for the composite outcome by enrollment sites. Table S3: Raw numbers for the composite outcome by subgroups. Table S4: Charlson Comorbidity Index components of study participants by BWV. Table S5: Cox proportional hazards regression of BWV for the fatal and non-fatal CV events in various subgroups. Table S6: Cox proportional hazards regression of BWV for all-cause mortality in various subgroups. Table S7: Multinomial logistic regression of BWV for longitudinal BW change. Table S8: Cox proportional hazards regression of BMI in addition to BWV for the outcomes. Figure S1: Scatter plot for the correlations of BW slope with BWV.
