# Supplementary material for: Association of Body Weight Variability with Adverse Cardiovascular Outcomes in Patients with Pre-Dialysis Chronic Kidney Disease
Source: Nutrients. 2021 Sep 26;13(10):3381. doi: 10.3390/nu13103381 (PMC8539704; doi:10.3390/nu13103381)
Supplement: Supplementary file 1 [file nutrients-13-03381-s001.zip › nutrients-1390086-supplementary.pdf]

Supplementary Materials

Association of body weight variability with adverse cardiovascular outcomes in patients with pre-dialysis chronic kidney disease

Sang Heon Suh, M.D., Ph.D.[1], Tae Ryom Oh, M.D., Ph.D.[1], Hong Sang Choi, M.D., Ph.D.[1], Chang Seong Kim, M.D., Ph.D.[1], Eun Hui Bae, M.D., Ph.D.[1], Sue K. Park, M.D, Ph.D.[2,3,4], Yong-Soo Kim, M.D., Ph.D.[5], Yeong Hoon Kim, M.D., Ph.D.[6], Kyu Hun Choi, M.D., Ph.D.[7], Kook-Hwan Oh, M.D., Ph.D.[8], Seong Kwon Ma*, M.D., Ph.D.[1], and Soo Wan Kim*, M.D., Ph.D.[1], on behalf of the Korean Cohort Study for Outcomes in Patients With Chronic Kidney Disease (KNOW-CKD) Investigators

[1]Department of Internal Medicine, Chonnam National University Medical School and Chonnam National University Hospital, Gwangju, Korea
[2]Department of Preventive Medicine, Seoul National University College of Medicine, Seoul, Korea
[3]Cancer Research Institute, Seoul National University, Seoul, Korea
[4]Integrated Major in Innovative Medical Science, Seoul National University College of Medicine, Seoul, Korea
[5]Department of Internal Medicine, College of Medicine, Catholic University of Korea, Seoul, Korea
[6]Department of Internal Medicine, College of Medicine, Busan Paik Hospital, Inje University, Busan, Korea.
[7]Department of Internal Medicine, College of Medicine, Institute of Kidney Disease Research, Yonsei University, Seoul, Korea
[8]Department of Internal Medicine, Seoul National University Hospital, Seoul, Korea

Running title: BW variability and CV outcomes in CKD

Corresponding authors

*Seong Kwon Ma, MD, PhD. Department of Internal Medicine, Chonnam National University Medical School, 42 Jebongro, Gwangju 61469, Korea, Tel: +82-62-220-6579, Fax: +82-62-225-8578, Email: drmsk@hanmail.net

*Soo Wan Kim, MD, PhD. Department of Internal Medicine, Chonnam National University Medical School, 42 Jebongro, Gwangju 61469, Korea, Tel: +82-62-220-6271, Fax: +82-62-225-8578, Email: skimw@chonnam.ac.kr

<u>Table of Contents</u>

Table S1. Baseline characteristics of study participants by BW slope
Table S2. Raw numbers for the composite outcome by enrollment sites
Table S3. Raw numbers for the composite outcome by subgroups
Table S4. Charlson Comorbidity Index components of study participants by BWV
Table S5. Cox proportional hazards regression of BWV for the fatal and non-fatal CV events in various subgroups
Table S6. Cox proportional hazards regression of BWV for all-cause mortality in various subgroups
Table S7. Multinomial logistic regression of BWV for longitudinal BW change
Table S8. Cox proportional hazards regression of BMI in addition to BWV for the outcomes
[illegible]

Table S1. Baseline characteristics of study participants by BW slope

	BW change			
	Decrease	Maintenance	Increase	*P* value
**Annual rate of BW change (kg/year)**	-1.540 ± 1.341	-0.014 ± 0.290	1.360 ± 1.226	< 0.001
**Age (year)**	55.184 ± 12.138	54.323 ± 11.507	50.558 ± 12.621	< 0.001
**Male**	292 (61.7)	249 (59.0)	292 (62.9)	0.322
**Charlson comorbidity index**				0.005
**0 – 3**	325 (68.7)	724 (77.8)	334 (72.0)	
**≥ 4**	148 (31.3)	206 (22.2)	130 (28.0)	
**DM**	184 (38.9)	243 (26.1)	165 (35.6)	< 0.001
**CAD**	33 (7.0)	45 (4.8)	29 (6.3)	0.564
**Arrhythmia**	22 (4.7)	14 (1.5)	8 (1.7)	< 0.001
**Current smoking**	76 (16.1)	147 (15.8)	66 (14.2)	0.684
**BMI (kg/m^2)**	25.502 ± 3.411	24.216 ± 3.106	24.335 ± 3.622	< 0.001
**WC (cm)**	84.533 ± 21.573	80.728 ± 23.086	83.630 ± 20.785	0.004
**SBP (mmHg)**	127.159 ± 17.202	126.123 ± 15.172	126.970 ± 15.864	0.432
**DBP (mmHg)**	76.233 ± 11.860	76.486 ± 10.778	77.183 ± 10.985	0.388
**Medications**				
**ACEi/ARBs**	407 (86.0)	808 (86.9)	403 (86.9)	0.901
**Diuretics**	169 (35.7)	258 (27.7)	147 (31.7)	0.008
**Number of anti-HTN drugs ≥ 3**	151 (31.9)	228 (24.5)	141 (30.4)	0.005
**Laboratory findings**				
**Hemoglobin (g/dL)**	12.727 ± 1.984	13.138 ± 1.888	12.984 ± 2.080	0.001
**Albumin (g/dL)**	4.136 ± 0.429	4.245 ± 0.349	4.234 ± 0.387	< 0.001
**Total cholesterol (mg/dL)**	174.339 ± 383.063	173.124 ± 37.378	174.365 ± 38.011	0.782
**LDL cholesterol (mg/dL)**	96.524 ± 332.109	95.456 ± 30.116	97.562 ± 30.129	0.477
**HDL cholesterol (mg/dL)**	48.615 ± 15.045	50.459 ± 15.797	48.732 ± 14.502	0.044
**Triglyceride (mg/dL)**	168.609 ± 102.519	151.492 ± 94.493	155.793 ± 102.683	0.009
**Fasting glucose (mg/dL)**	113.837 ± 43.259	107.142 ± 31.613	111.446 ± 45.172	0.006
**hsCRP (mg/dL)**	0.8000 [0.300, 2.000]	0.545 [0.200, 1.500]	0.600 [0.200, 1.500]	0.307
**25(OH) vitamin D (ng/dL)**	17.866 ± 7.749	18.640 ± 8.179	16.974 ± 6.939	0.001
**24-hr urine protein (mg/dL)**	738.900 [292.450]	430.400 [152.350, 1085.250]	490.900 [138.000, 1345.550]	< 0.001
**Urine ACR (mg/g Cr)**	494.728 [162.643, 1303.109]	270.194 [49.640, 741.250]	255.451 [60.281, 843.487]	< 0.001
**eGFR (mL/min./1.73m^2)**	46.196 ± 29.051	54.223 ± 28.237	55.637 ± 31.307	< 0.001

Table S1. (Continued)

	BW change			
	Decrease	Maintenance	Increase	*P* value
**CKD stages**				< 0.001
**Stage 1**	62 (13.1)	159 (17.1)	95 (20.5)	
**Stage 2**	65 (13.7)	218 (23.4)	97 (20.9)	
**Stage 3a**	73 (15.4)	168 (18.1)	90 (19.4)	
**Stage 3b**	114 (24.1)	216 (23.2)	83 (17.9)	
**Stage 4**	130 (27.5)	150 (16.1)	85 (18.3)	
**Stage 5**	29 (6.1)	19 (2.0)	14 (3.0)	

Note: Values for categorical variables are given as number (percentage); values for continuous variables, as mean ± standard deviation or median [interquartile range].

Abbreviations: ACEi, angiotensin-converting enzyme inhibitor; ACR, albumin-to-creatinine ratio; ARB, angiotensin receptor blocker; BMI, body mass index; CAD, coronary artery disease; CCI, Charlson comorbidity index; CKD, chronic kidney disease; DBP, diastolic blood pressure; DM, diabetes mellitus; eGFR, estimated glomerular filtration rate; hsCRP, high-sensitivity C-reactive protein; SBP, systolic blood pressure; WC, waist circumference.

Table S2. Raw numbers for the composite outcome by enrollment sites

	Site 1	Site 2	Site 3	Site 4	Site 5	Site 6	Site 7	Site 8	Site 9	P value
**Event cases**	17	8	19	17	10	8	14	48	22	0.592
**Total cases**	125	112	199	164	137	109	199	588	234	

Note: Values are given as raw number.

Table S3. Raw numbers for the composite outcome by subgroups

		Event cases	Total cases
**All subjects**	**Low BWV**	30	496
	**Moderate BWV**	83	934
	**High BWV**	50	464
**Age < 60 years**	**Low BWV**	13	297
	**Moderate BWV**	32	606
	**High BWV**	21	316
**Age ≥ 60 years**	**Low BWV**	17	172
	**Moderate BWV**	51	328
	**High BWV**	29	148
**Male**	**Low BWV**	22	278
	**Moderate BWV**	58	556
	**High BWV**	35	299
**Female**	**Low BWV**	8	191
	**Moderate BWV**	25	378
	**High BWV**	15	165
**CCI ≤ 3**	**Low BWV**	12	360
	**Moderate BWV**	49	710
	**High BWV**	23	313
**CCI ≥ 4**	**Low BWV**	18	109
	**Moderate BWV**	34	224
	**High BWV**	27	151
**DM (-)**	**Low BWV**	13	344
	**Moderate BWV**	46	654
	**High BWV**	19	277
**DM (+)**	**Low BWV**	17	125
	**Moderate BWV**	37	280
	**High BWV**	31	187
**BMI < 25 (kg/m^2)**	**Low BWV**	20	300
	**Moderate BWV**	52	561
	**High BWV**	24	215
**BMI ≥ 25 (kg/m^2)**	**Low BWV**	10	169
	**Moderate BWV**	31	373
	**High BWV**	26	249

Table S3. (Continued)

		Event cases	Total cases
**Diuretics (-)**	**Low BWV**	17	320
	**Moderate BWV**	57	668
	**High BWV**	27	305
**Diuretics (+)**	**Low BWV**	13	149
	**Moderate BWV**	26	266
	**High BWV**	23	159
**Number of anti-HTN drugs ≤2**	**Low BWV**	24	354
	**Moderate BWV**	53	690
	**High BWV**	31	303
**Number of anti-HTN drugs ≥ 3**	**Low BWV**	6	115
	**Moderate BWV**	30	244
	**High BWV**	19	161
**eGFR ≥ 45 mL/min./1.73m^2**	**Low BWV**	9	216
	**Moderate BWV**	39	509
	**High BWV**	20	238
**eGFR < 45 mL/min./1.73m^2**	**Low BWV**	21	226
	**Moderate BWV**	44	425
	**High BWV**	30	253
**Random urine ACR < 300 mg/g**	**Low BWV**	10	220
	**Moderate BWV**	49	456
	**High BWV**	19	211
**Random urine ACR ≥ 300 mg/g**	**Low BWV**	19	235
	**Moderate BWV**	35	437
	**High BWV**	30	238

Note: Values are given as raw number.
Abbreviations: ACR, Albumin-to-creatinine ratio; BMI, body mass index; CCI, Charlson comorbidity index; CI, confidence interval; DM, diabetes mellitus; HTN, hypertension; eGFR, estimated glomerular filtration rate.

Table S4. Charlson Comorbidity Index components of study participants by BWV

	BWV			*P* value
	Low	Moderate	High	
Myocardial infarction	8 (1.7)	10 (1.1)	7 (1.5)	0.581
Congestive Heart Failure	3 (0.6)	8 (0.9)	8 (1.7)	0.201
Peripheral vascular disease	11 (2.3)	37 (4.0)	17 (3.7)	0.288
Cerebrovascular disease	23 (4.9)	43 (4.6)	41 (8.8)	0.004
Dementia	1 (0.2)	0 (0.0)	0 (0.0)	0.225
COPD	1 (0.2)	7 (0.7)	1 (0.2)	0.248
Connective tissue disease	26 (5.5)	56 (6.0)	32 (6.9)	0.676
Peptic ulcer disease	8 (1.7)	18 (1.9)	7 (1.5)	0.849
Mild liver disease	10 (2.1)	19 (2.0)	15 (3.2)	0.355
Moderate-to-severe liver disease or Liver cirrhosis	0 (0.0)	2 (0.2)	0 (0.0)	0.368
Diabetes without complication	20 (4.3)	53 (5.7)	32 (6.2)	0.217
Diabetes with organ damage	105 (22.4)	226 (24.2)	155 (33.4)	< 0.001
Hemiplegia	3 (0.6)	2 (0.2)	4 (0.9)	0.219
Moderate-to-severe renal disease	348 (74.2)	626 (67.0)	320 (69.0)	0.022
Any tumor within last 5 years	0 (0.0)	0 (0.0)	0 (0.0)	
Lymphoma	0 (0.0)	0 (0.0)	0 (0.0)	
Leukemia	0 (0.0)	0 (0.0)	0 (0.0)	
Metastatic solid tumor	0 (0.0)	0 (0.0)	0 (0.0)	
AIDS	0 (0.0)	0 (0.0)	0 (0.0)	

Note: Values for categorical variables are given as number (percentage).

Abbreviations: AIDS, acquired immunodeficiency syndrome; COPD, chronic obstructive pulmonary disease.

Table S5. Cox proportional hazards regression of BWV for the fatal and non-fatal CV events in various subgroups

		Unadjusted		Adjusted	
		HR (95%CIs)	*P* value	HR (95%CIs)	*P* value
**Age < 60 years**	**Low BWV**	1.810 (0.816, 4.011)	0.144	0.573 (0.174, 1.888)	0.360
	**Moderate BWV**	Reference		Reference	
	**High BWV**	1.689 (0.889, 3.208)	0.109	0.747 (0.252, 2.217)	0.599
**Age ≥ 60 years**	**Low BWV**	1.137 (0.582, 2.221)	0.708	1.380 (0.586, 3.251)	0.462
	**Moderate BWV**	Reference		Reference	
	**High BWV**	2.181 (1.284, 3.704)	0.004	2.191 (1.054, 4.557)	0.036
**Male**	**Low BWV**	1.894 (0.986, 3.639)	0.055	1.770 (0.772, 4.059)	0.178
	**Moderate BWV**	Reference		Reference	
	**High BWV**	1.529 (0.944, 2.476)	0.084	1.865 (1.020, 3.412)	0.043
**Female**	**Low BWV**	0.713 (0.300, 1.695)	0.444	0.361 (0.074, 1.770)	0.209
	**Moderate BWV**	Reference		Reference	
	**High BWV**	2.631 (1.206, 5.741)	0.015	0.615 (0.153, 2.476)	0.493
**CCI ≤ 3**	**Low BWV**	0.974 (0.454, 2.087)	0.946	0.915 (0.307, 2.728)	0.874
	**Moderate BWV**	Reference		Reference	
	**High BWV**	2.239 (1.230, 4.075)	0.008	1.901 (0.810, 4.460)	0.140
**CCI ≥ 4**	**Low BWV**	1.851 (0.916, 3.742)	0.086	4.447 (1.547, 12.786)	0.006
	**Moderate BWV**	Reference		Reference	
	**High BWV**	1.560 (0.878, 2.770)	0.129	1.962 (0.908, 4.238)	0.086
**DM (-)**	**Low BWV**	1.261 (0.604, 2.630)	0.537	1.626 (0.576, 4.587)	0.359
	**Moderate BWV**	Reference		Reference	
	**High BWV**	1.946 (1.000, 3.788)	0.050	1.548 (0.575, 4.165)	0.387
**DM (+)**	**Low BWV**	1.347 (0.661, 2.473)	0.412	2.099 (0.699, 6.300)	0.186
	**Moderate BWV**	Reference		Reference	
	**High BWV**	1.687 (0.989, 2.878)	0.055	2.300 (1.147, 4.612)	0.019
**BMI < 25 (kg/m^2)**	**Low BWV**	1.484 (0.789, 2.789)	0.220	2.131 (0.924, 4.914)	0.076
	**Moderate BWV**	Reference		Reference	
	**High BWV**	1.963 (1.109, 3.475)	0.021	1.923 (0.900, 4.113)	0.092
**BMI ≥ 25 (kg/m^2)**	**Low BWV**	0.996 (0.407, 2.436)	0.993	1.138 (0.284, 4.565)	0.855
	**Moderate BWV**	Reference		Reference	
	**High BWV**	1.602 (0.880, 2.914)	0.123	3.048 (1.181, 7.869)	0.021

Table S5. (continued)

		Unadjusted		Adjusted	
		HR (95%CIs)	*P* value	HR (95%CIs)	*P* value
**Diuretics (-)**	**Low BWV**	1.716 (0.872, 3.376)	0.118	2.286 (0.938, 5.572)	0.069
	**Moderate BWV**	Reference		Reference	
	**High BWV**	3.041 (1.659, 5.573)	< 0.001	2.515 (1.165, 5.428)	0.019
**Diuretics (+)**	**Low BWV**	0.997 (0.448, 2.221)	0.995	0.677 (0.201, 2.280)	0.529
	**Moderate BWV**	Reference		Reference	
	**High BWV**	1.365 (0.747, 2.496)	0.312	1.106 (0.469, 2.608)	0.817
**Number of anti-HTN drugs ≤2**	**Low BWV**	1.332 (0.738, 2.406)	0.342	1.715 (0.811, 3.629)	0.158
	**Moderate BWV**	Reference		Reference	
	**High BWV**	1.637 (0.981, 2.732)	0.059	1.615 (0.817, 3.194)	0.168
**Number of anti-HTN drugs ≥ 3**	**Low BWV**	0.986 (0.339, 2.870)	0.980	0.889 (0.118, 6.728)	0.909
	**Moderate BWV**	Reference		Reference	
	**High BWV**	2.504 (1.264, 4.963)	0.009	7.993 (2.145, 29.779)	0.002
**eGFR ≥ 45 mL/min./1.73m^2**	**Low BWV**	1.651 (0.630, 4.327)	0.308	0.285 (0.061, 1.340)	0.112
	**Moderate BWV**	Reference		Reference	
	**High BWV**	1.976 (1.063, 3.671)	0.031	2.142 (0.920, 4.991)	0.077
**eGFR < 45 mL/min./1.73m^2**	**Low BWV**	1.201 (0.648, 2.224)	0.561	1.668 (0.734, 3.793)	0.222
	**Moderate BWV**	Reference		Reference	
	**High BWV**	1.847 (1.073, 3.179)	0.027	2.195 (1.051, 4.582)	0.036
**Random urine ACR < 300 mg/g**	**Low BWV**	1.583 (0.723, 3.464)	0.251	2.915 (0.982, 8.648)	0.054
	**Moderate BWV**	Reference		Reference	
	**High BWV**	2.111 (1.130, 3.944)	0.019	2.219 (0.921, 5.348)	0.076
**Random urine ACR ≥ 300 mg/g**	**Low BWV**	1.061 (0.536, 2.099)	0.865	1.321 (0.506, 3.445)	0.570
	**Moderate BWV**	Reference		Reference	
	**High BWV**	1.554 (0.890, 2.715)	0.121	1.932 (0.913, 4.091)	0.085

Note: Models were adjusted for age, gender, Charlson comorbidity index, history of DM, smoking history, BMI, SBP, DBP, Medications (ACEi/ARBs, diuretics, Number of antihypertensive drugs), hemoglobin, albumin, HDL-cholesterol, triglycerides, fasting serum glucose, hs-CRP, 25(OH) vitamin D levels, eGFR, and 24-hour urine protein.
Abbreviations: ACR, Albumin-to-creatinine ratio; BMI, body mass index; CCI, Charlson comorbidity index; CI, confidence interval; DM, diabetes mellitus; HTN, hypertension; eGFR, estimated glomerular filtration rate.

Table S6. Cox proportional hazards regression of BWV for all-cause mortality in various subgroups

		Unadjusted		Adjusted	
		HR (95%CIs)	*P* value	HR (95%CIs)	*P* value
**Age < 60 years**	**Low BWV**	0.994 (0.316, 3.124)	0.992	0.508 (0.139, 1.862)	0.307
	**Moderate BWV**	Reference		Reference	
	**High BWV**	1.101 (0.407, 2.980)	0.850	0.888 (0.278, 2.843)	0.888
**Age ≥ 60 years**	**Low BWV**	0.960 (0.461, 1.999)	0.912	0.998 (0.466, 2.138)	0.995
	**Moderate BWV**	Reference		Reference	
	**High BWV**	2.182 (1.238, 3.845)	0.007	2.438 (1.324, 4.490)	0.004
**Male**	**Low BWV**	1.061 (0.511, 2.205)	0.873	0.950 (0.446, 2.025)	0.895
	**Moderate BWV**	Reference		Reference	
	**High BWV**	1.625 (0.914, 2.890)	0.098	2.039 (1.093, 3.807)	0.025
**Female**	**Low BWV**	0.984 (0.308, 3.137)	0.978	1.064 (0.316, 3.582)	0.920
	**Moderate BWV**	Reference		Reference	
	**High BWV**	1.780 (0.702, 4.511)	0.224	1.374 (0.451, 4.186)	0.576
**CCI ≤ 3**	**Low BWV**	0.878 (0.351, 2.201)	0.782	0.736 (0.308, 2.058)	0.638
	**Moderate BWV**	Reference		Reference	
	**High BWV**	1.733 (0.855, 3.512)	0.127	2.636 (1.227, 5.662)	0.013
**CCI ≥ 4**	**Low BWV**	1.113 (0.480, 2.578)	0.804	0.983 (0.393, 2.461)	0.971
	**Moderate BWV**	Reference		Reference	
	**High BWV**	1.311 (0.662, 2.595)	0.437	1.556 (0.714, 3.390)	0.266
**DM (-)**	**Low BWV**	1.306 (0.586, 2.910)	0.513	1.022 (0.441, 2.370)	0.959
	**Moderate BWV**	Reference		Reference	
	**High BWV**	1.408 (0.649, 3.053)	0.386	1.701 (0.736, 3.933)	0.214
**DM (+)**	**Low BWV**	0.761 (0.282, 2.051)	0.589	0.682 (0.245, 1.898)	0.463
	**Moderate BWV**	Reference		Reference	
	**High BWV**	1.580 (0.829, 3.011)	0.164	1.707 (0.815, 3.576)	0.156
**BMI < 25 (kg/m^2)**	**Low BWV**	1.050 (0.481, 2.293)	0.903	0.902 (0.300, 2.713)	0.855
	**Moderate BWV**	Reference		Reference	
	**High BWV**	2.361 (1.245, 4.479)	0.009	3.413 (1.372, 8.491)	0.008
**BMI ≥ 25 (kg/m^2)**	**Low BWV**	0.972 (0.353, 2.677)	0.956	0.846 (0.293, 2.448)	0.758
	**Moderate BWV**	Reference		Reference	
	**High BWV**	1.160 (0.543, 2.479)	0.701	1.129 (0.476, 2.677)	0.783

Table S6. (continued)

		Unadjusted		Adjusted	
		HR (95%CIs)	*P* value	HR (95%CIs)	*P* value
**Diuretics (-)**	**Low BWV**	1.334 (0.599, 2.971)	0.480	1.203 (0.524, 2.763)	0.663
	**Moderate BWV**	Reference		Reference	
	**High BWV**	1.812 (0.901, 3.645)	0.095	1.993 (0.941, 4.218)	0.071
**Diuretics (+)**	**Low BWV**	0.684 (0.253, 1.848)	0.454	0.733 (0.251, 2.139)	0.570
	**Moderate BWV**	Reference		Reference	
	**High BWV**	1.435 (0.723, 2.849)	0.302	1.574 (0.682, 3.631)	0.288
**Number of anti-HTN drugs ≤2**	**Low BWV**	1.122 (0.511, 2.466)	0.774	1.064 (0.474, 2.389)	0.880
	**Moderate BWV**	Reference		Reference	
	**High BWV**	1.847 (0.945, 3.609)	0.073	1.773 (0.854, 3.681)	0.124
**Number of anti-HTN drugs ≥ 3**	**Low BWV**	0.930 (0.340, 2.542)	0.887	0.674 (0.208, 2.189)	0.512
	**Moderate BWV**	Reference		Reference	
	**High BWV**	1.267 (0.617, 2.600)	0.519	1.837 (0.770, 4.382)	0.170
**eGFR ≥ 45 mL/min./1.73m^2**	**Low BWV**	0.779 (0.165, 3.672)	0.753	0.624 (0.123, 3.161)	0.569
	**Moderate BWV**	Reference		Reference	
	**High BWV**	2.623 (1.035, 6.647)	0.042	2.459 (0.784, 7.709)	0.123
**eGFR < 45 mL/min./1.73m^2**	**Low BWV**	0.959 (0.486, 1.892)	0.903	0.906 (0.442, 1.855)	0.786
	**Moderate BWV**	Reference		Reference	
	**High BWV**	1.449 (0.808, 2.599)	0.213	1.409 (0.743, 2.673)	0.294
**Random urine ACR < 300 mg/g**	**Low BWV**	1.087 (0.451, 2.623)	0.853	0.989 (0.385, 2.543)	0.982
	**Moderate BWV**	Reference		Reference	
	**High BWV**	1.857 (0.915, 3.768)	0.086	2.580 (1.177, 5.660)	0.018
**Random urine ACR ≥ 300 mg/g**	**Low BWV**	1.025 (0.426, 2.463)	0.956	1.030 (0.403, 2.631)	0.950
	**Moderate BWV**	Reference		Reference	
	**High BWV**	1.546 (0.769, 3.112)	0.222	1.679 (0.730, 3.859)	0.222

Note: Models were adjusted for age, gender, Charlson comorbidity index, history of DM, smoking history, BMI, SBP, DBP, Medications (ACEi/ARBs, diuretics, Number of antihypertensive drugs), hemoglobin, albumin, HDL-cholesterol, triglycerides, fasting serum glucose, hs-CRP, 25(OH) vitamin D levels, eGFR, and 24-hour urine protein.
Abbreviations: ACR, Albumin-to-creatinine ratio; BMI, body mass index; CCI, Charlson comorbidity index; CI, confidence interval; DM, diabetes mellitus; HTN, hypertension; eGFR, estimated glomerular filtration rate.

Table S7. Multinomial logistic regression of BWV for longitudinal BW change

		BW decrease *vs.* BW maintenance		BW increase *vs.* BW maintenance	
		**OR (95%CIs)**	***P* value**	**OR (95%CIs)**	***P* value**
**Unadjusted**	**BWV, low**	0.412 (0.304, 0.558)	< 0.001	0.481 (0.356, 0.649)	< 0.001
	**BWV, moderate**	Reference		Reference	
	**BWV, high**	2.598 (1.966, 3.432)	< 0.001	2.907 (2.198, 3.846)	< 0.001
**Adjusted**	**BWV, low**	0.429 (0.300, 0.614)	< 0.001	0.541 (0.384, 0.763)	< 0.001
	**BWV, moderate**	Reference		Reference	
	**BWV, high**	2.310 (1.661, 3.213)	< 0.001	2.642 (1.912, 3.652)	< 0.001

Note: Models were adjusted for age, gender, Charlson comorbidity index, history of DM, smoking history, BMI, SBP, DBP, Medications (ACEi/ARBs, diuretics, number of antihypertensive drugs), hemoglobin, albumin, HDL-cholesterol, triglycerides, fasting serum glucose, hs-CRP, 25(OH) vitamin D levels, eGFR, and 24-hour urine protein.

Abbreviations: ACR, Albumin-to-creatinine ratio; BMI, body mass index; CCI, Charlson comorbidity index; CI, confidence interval; DM, diabetes mellitus; HTN, hypertension; eGFR, estimated glomerular filtration rate.

Table S8. Cox proportional hazards regression of BMI in addition to BWV for the outcomes

		Unadjusted		Adjusted	
		HR (95%CIs)	*P* value	HR (95%CIs)	*P* value
**Composite outcome**	**Low BWV**	1.236 (0.743, 2.056)	0.416	1.455 (0.795, 2.663)	0.224
	**Moderate BWV**	Reference		Reference	
	**High BWV, BMI 20 - 24.9 kg/m^2**	1.967 (1.205, 3.210)	0.007	1.482 (0.808, 2.721)	0.204
	**High BWV, BMI < 20 or ≥ 25 kg/m^2**	1.592 (0.917, 2.766)	0.099	2.149 (1.107, 4.173)	0.024
**Fatal and non-fatal CV events**	**Low BWV**	1.281 (0.771, 2.131)	0.339	1.602 (0.884, 2.902)	0.120
	**Moderate BWV**	Reference		Reference	
	**High BWV, BMI 20 - 24.9 kg/m^2**	1.998 (1.225, 3.258)	0.006	1.582 (0.873, 2.868)	1.582
	**High BWV, BMI < 20 or ≥ 25 kg/m^2**	1.636 (0.942, 2.842)	0.081	2.286 (1.180, 4.426)	0.014
**All-cause mortality**	**Low BWV**	1.008 (0.543, 1.869)	0.980	0.933 (0.493, 1.759)	0.831
	**Moderate BWV**	Reference		Reference	
	**High BWV, BMI 20 - 24.9 kg/m^2**	1.425 (0.768, 2.642)	0.262	1.618 (0.810, 3.232)	0.173
	**High BWV, BMI < 20 or ≥ 25 kg/m^2**	2.078 (1.138, 3.796)	0.017	2.124 (1.101, 4.095)	0.025

Note: Models were adjusted for age, gender, Charlson comorbidity index, history of DM, smoking history, BMI, SBP, DBP, Medications (ACEi/ARBs, diuretics, Number of antihypertensive drugs), hemoglobin, albumin, HDL-cholesterol, triglycerides, fasting serum glucose, hs-CRP, 25(OH) vitamin D levels, eGFR, and 24-hour urine protein.

Abbreviations: BWV, body weight variability; CV, cardiovascular; CI, confidence interval.

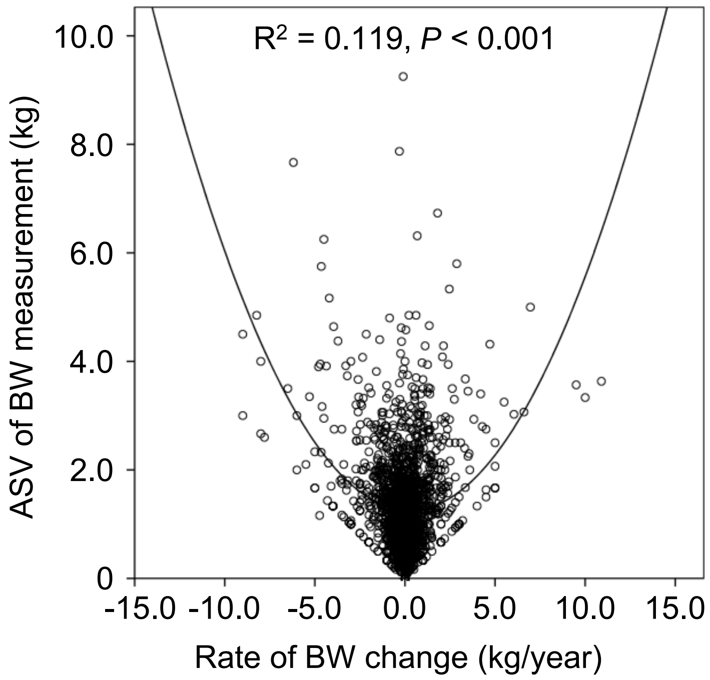

Figure S1. Scatter plot for the correlations of BW slope with BWV

Abbreviations: ASV, average successive variability; BW, body weight; BWV, body weight variability
